# Integrating Beach Monitoring and Satellite Telemetry to Estimate Loggerhead Clutch Frequency in Brazil

**DOI:** 10.3390/ani16020320

**Published:** 2026-01-21

**Authors:** Paulo Hunold Lara, Gustavo Stahelin, Maria Ângela Marcovaldi, Alexsandro Santana dos Santos, Yonat Swimmer, Milagros López Mendilaharsu

**Affiliations:** 1 Fundação Projeto Tamar, Rua Rubens Guelli 134, sl 307, Salvador 41815-135, BA, Brazil; paulo.lara@tamar.org.br (P.H.L.); gustavo@tamar.org.br (G.S.); neca@tamar.org.br (M.Â.M.); alex@tamar.org.br (A.S.d.S.); 2 Pacific Islands Fisheries Science Center, NOAA Fisheries, Honolulu, HI 96818, USA

**Keywords:** *Caretta caretta*, satellite tracking, beach monitoring, clutch frequency, reproductive output, conservation strategies, nesting beaches, Brazil

## Abstract

Accurate clutch-frequency estimates are essential for assessing population abundance and reproductive output in sea turtles. Traditional nighttime beach-monitoring approaches, however, often underestimate clutch frequency by missing nesting events occurring outside patrolled beaches. Here, we integrated long-term beach monitoring (2009–2016) with satellite telemetry to estimate the clutch frequency of loggerhead turtles (*Caretta caretta*) nesting at Praia do Forte, Bahia, Brazil. A total of 593 females were identified along a 5 km monitored beach segment, and transient individuals represented 42.4% ± 3.9 SD of seasonal records. A 2-year remigration interval was the most frequent. The observed clutch frequency (OCF) averaged 3.1 ± 1.2 SD clutches per female, while the estimated clutch frequency based on beach monitoring alone (ECF_BM) averaged 3.9 ± 1.5 SD. For the subset of satellite-tracked females (n = 12), integration of residency length derived from telemetry increased the estimate to 5.6 ± 0.7 SD clutches per female (ECF_BMST). Statistical comparisons confirmed significant differences among estimation methods. These findings align with previous studies, demonstrating that clutch frequency is substantially underestimated when relying solely on beach monitoring. Incorporating satellite telemetry, therefore, provides a more accurate assessment of reproductive output and has important implications for population modelling and the conservation of loggerhead turtles in Brazil.

## Simple Summary

Although conservation status varies among species, most sea turtle species are globally threatened. Estimating their population size is challenging because only adult females come ashore to nest during the breeding season. Knowing how many females nest each year is essential for understanding population trends and guiding effective conservation measures. Sea turtle nesting female abundance is often derived from nest counts and clutch frequency (CF). However, CF based on nighttime patrols alone can be inaccurate, as some nesting events are often missed or occur outside surveyed areas. In the current study, we combined traditional nighttime beach monitoring with satellite tracking of loggerhead turtles nesting in Bahia, Brazil, to improve the accuracy of CF estimates. Satellite tracking data allowed estimation of nesting events missed during beach patrols, indicating that females lay more nests per season than previously estimated. Consequently, earlier assessments based only on beach monitoring may have overestimated the number of breeding females. This integrated approach provides a more reliable estimate of reproductive parameters, which are extremely important for population abundance assessments of loggerhead turtles in Brazil.

## 1. Introduction

Sea turtles are globally recognized as species of conservation concern, with several populations classified as threatened due to persistent threats such as fisheries bycatch, habitat loss, and climate change [1]. Sea turtle conservation relies heavily on estimates of population abundance, often derived from nest counts collected during beach monitoring. These estimates are crucial for assessing the status and trends of marine turtle populations and informing conservation management strategies [2,3].

Clutch frequency (CF), the number of clutches a female turtle lays per nesting season, is a critical demographic parameter for both population modelling and abundance assessments, as it helps estimate the number of females based on observed nests [2]. To estimate the number of breeding females in a given population during a specific season, researchers commonly divide the total number of nests observed across all nesting beaches by the mean CF [4,5]. However, estimating clutch frequency from beach monitoring alone has been shown to underestimate this number, due to incomplete detection of nesting events by a given female [6,7,8]. For example, some female loggerheads have been recorded nesting up to eight or more times in a single season [9,10], while others have deposited nests over a range of more than 100 km [11], increasing the likelihood that not all nesting events from each individual are recorded by beach-based monitoring programs.

Recent studies have shown that combining beach monitoring with satellite telemetry enhances the accuracy of CF estimates [6,9,12]. This approach detects nesting events missed during beach monitoring and provides departure time data, resulting in more complete and robust information. For instance, satellite telemetry has revealed that hawksbill turtles nesting in Brazil and green turtles nesting on Ascension Island lay almost twice as many clutches as previously thought based on beach monitoring alone [6,7]. This has profound implications for population abundance estimates, which may require urgent reassessment.

Moreover, the variability in individual nesting behavior and the stochastic nature of nesting events further complicate these estimates, suggesting that reliance on mean clutch frequency values may be insufficient [13]. Thus, integrating satellite telemetry with traditional beach monitoring provides a more reliable approach to determining clutch frequency. Historically, reliable CF data have been lacking for loggerhead turtle populations in Brazil, particularly in key nesting areas, such as the coast in the state of Bahia. Due to the absence of this parameter, earlier studies relied on CF estimates derived from loggerhead nesting populations in other countries [14].

In the current study, we integrated nighttime capture-mark-recapture with satellite telemetry data to calculate a more precise clutch frequency (CF) estimate for loggerhead turtles nesting on the beach at Praia do Forte in Bahia, one of Brazil’s most important nesting areas for this species [14].

## 2. Materials and Methods

### 2.1. Study Area

**Figure 1:**
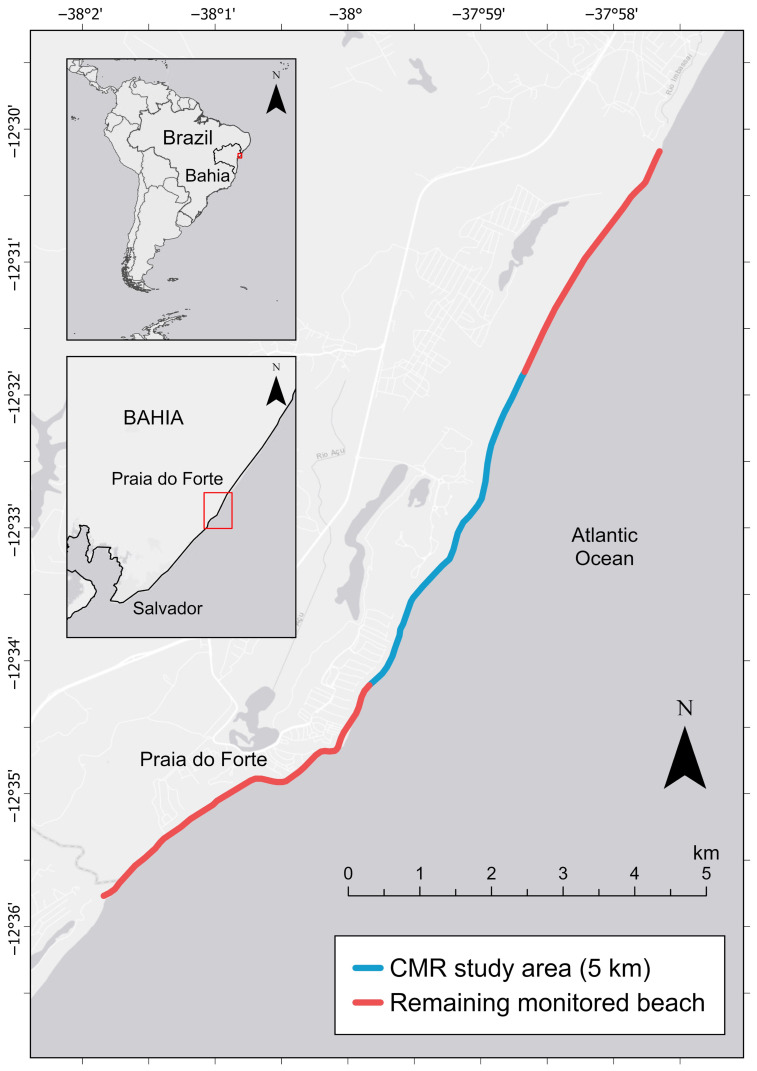
Praia do Forte monitored beach, showing the 5 km capture–mark–recapture (CMR) study section in which 65% of nesting activity is concentrated (blue) and the remaining daytime-only monitored beach section (red).

Located at one of the major sea turtle rookeries in Brazil, Praia do Forte beach in the state of Bahia stretches along 14 km of the coastline (12°35′45″–12°30′9.95″ S and 38°1′46″–37°57′38″ W) and hosts nests of loggerheads (*Caretta caretta*), hawksbills (*Eretmochelys imbricata*), olive Ridley (*Lepidochelys olivacea*), and green turtles (*Chelonia mydas*) (Figure 1) [15]. Loggerhead nesting season in Praia do Forte lasts from September to April; however, 90% of nesting activity is concentrated between October and February. Within the Praia do Forte beach, 65% of the nests occur along a 5 km segment (Figure 1).

### 2.2. Field Methods

The 14 km stretch of Praia do Forte has been patrolled daily in the daytime during the sea turtle nesting season since 1982 [15]. At the same time, a capture-mark-recapture (CMR) program has been conducted during night patrols of nesting females [15,16] with an inconsistent effort over the years (i.e., months, number of nights, and hours per night monitored). In 2008, we adjusted the night patrol effort to improve the accuracy of site-specific demographic parameters for loggerheads. We conducted a preliminary study in 2008 with daily nighttime surveys from 8 p.m. to 5 a.m. from October 15 to December, which coincides with the nesting peak [16]. As a result, we determined that 83.5% of the female encounters occurred between 8 p.m. and 2 a.m. We then conducted a nighttime tagging effort over seven consecutive nesting seasons from 2009/10 to 2015/16. Each season, from 1 October to 28 February, two researchers patrolled the 5 km stretch from 8 p.m. to 2 a.m. nightly. Nighttime patrols occurred even under rainy conditions, except when deemed unsafe (e.g., lightning or severe weather). As nesting seasons start and end on different calendar years, hereafter we refer to nesting seasons as the year in which they start: 2009/10 will be referred to as the 2009 nesting season.

After egg-laying, each turtle encountered was checked for the presence of tags, and if none were observed, it was tagged with Inconel tags (number 681 National Band and Tag Company, Newport, KY, USA) on both front flippers. Curved carapace length (CCL ± 0.1 cm) was measured with a flexible tape from notch (external border of the nuchal scale) to tip (external border of the supra-caudal scale) by one of the researchers present [17]. Animals were normally handled for biometrics and tagging for less than 5 min (estimated time, as handling time was not recorded).

### 2.3. Satellite Tracking and Analysis

A subset of 14 satellite tracked internesting intervals from 12 different females was selected from a larger study of loggerhead post-nesting migration (2013–2015). Two of the females were tracked over consecutive nesting seasons with a new satellite transmitter deployed each time.

**Figure 2:**
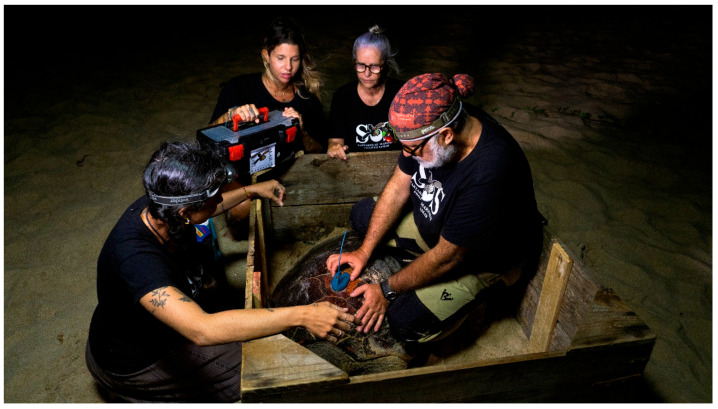
Attachment of a satellite transmitter to a loggerhead turtle in Praia do Forte, Brazil.

Turtles were fitted with satellite platform transmitter terminals (PTTs); Model SPOT (n = 11) and SPLASH (n = 3) from Wildlife Computers, Redmond, WA, USA (Figure 2). To attach the units, we restrained turtles in a wooden portable corral following nesting. The carapace was sanded and cleaned with isopropyl alcohol to prepare the surface for transmitter attachment. Transmitters were attached to the crown of the carapace using two-part epoxy glue (Tubolit MEP-301, Rezzolut Soluções Anticorrosivas Ltda, Duque de Caxias, RJ, Brazil) and covered with a layer of Micron Premium antifouling paint (AkzoNobel, Melbourne, Australia). Instrumented turtles were released at their capture sites within 4 h. The satellite tags’ duty cycle was to remain on for 24 h for the first 60 days, then switch to 24 h on and 48 h off to maximize battery duration. Haul-out data were not used to determine nesting events. Instead, the observed date of each female’s departure from the breeding area was used to extend her estimated residence time within the reproductive area. Locational data were obtained from the Argos satellite tracking system (www.argos-system.org, accessed on 1 February 2024) and filtered to retain reliable positions (LC 3, 2, 1, 0, A, and B) based on a maximum travel rate of 5 km h^−1^ following the filters applied in [18]. As satellite tracking data were used only to estimate departure date from nesting area, Argos data location errors are not of concern.

### 2.4. Clutch Frequency and Internesting Interval

We calculated the following: (1) the observed clutch frequency (OCF) through direct re-observation of the turtle on the beach during subsequent nesting emergences, (2) the estimated clutch frequency based on the beach monitoring data (ECF_BM) corrected by considering missed nests based on the mean internesting interval for this population (14.9 days [10]) and residency length (RL) as the last date the turtle was observed on the beach, and (3) the estimated clutch frequency by combining beach monitoring data and satellite telemetry (ECF_BMST). We used satellite telemetry data for ECF_BMST solely to estimate departure date (turtles depart their nesting site soon after depositing the final clutch; [19]) to obtain RL at the breeding ground [6] (Table 1). Females were included in the analysis if first encountered during the first half of the nesting season (until 1 January) to maximize the probability that selected turtles were nesting for the first time during the season. Considering the small sample size of satellite-tracked animals and to avoid biases with their inclusion, we have removed from the analyses animals identified as outliers following [20] (see Table 1).

Females observed nesting only once during the season (i.e., transients) were excluded from CF estimates. Because only a single nest was recorded for these individuals, it is not possible to determine whether that observation corresponds to the first, last, or an intermediate clutch, nor to infer any additional nesting event that may have occurred outside the patrolled beach stretch. As demonstrated previously, including transients can heavily bias mean clutch-frequency estimates downward [6], which was a concern given that our sampling effort was not designed to capture all individuals; therefore, only females documented nesting at least twice were considered in the analysis. A total of 349 records of transient animals were removed (mean = 50 animals per season; range 30–73).

We determined the remigration interval (RI) of adult females as the interval (in years) between two consecutive nesting seasons in which the same individual was recorded from 2008 to 2015. Animals observed nesting in more than one season were included in the OCF calculations for all years in which they were observed in more than one nesting event.

### 2.5. Statistical Comparisons

To evaluate differences among clutch-frequency estimates obtained using different monitoring approaches, we compared observed clutch frequency (OCF), estimated clutch frequency from beach monitoring alone (ECF_BM) from 2009 to 2015, and the combined estimate incorporating satellite telemetry (ECF_BMST) for the subset of satellite-tracked turtles. We applied a Kruskal–Wallis test to assess overall differences among methods. When significant, pairwise Wilcoxon signed-rank tests with Bonferroni correction were used for post-hoc comparisons. All statistical analyses were conducted using non-parametric tests, and significance was accepted at α = 0.05. Analyses were performed in R (version 4.2.3).

## 3. Results

Between the 2009 and 2016 nesting seasons, 593 turtles were identified along the 5 km beach stretch in Praia do Forte, Bahia (Figure 1). Each season, an average of 110 ± 33 SD females (range: 62–173) were encountered during night patrols. Among the females encountered, an average of 42.4% ± 3.9 SD were transient individuals observed nesting only once during the season.

Although the RI of individual females varies from 1 to 7 years, the 2-year RI was the most common, followed by the 3-year RI. Only one turtle was observed nesting in all eight nesting seasons.

**Figure 3:**
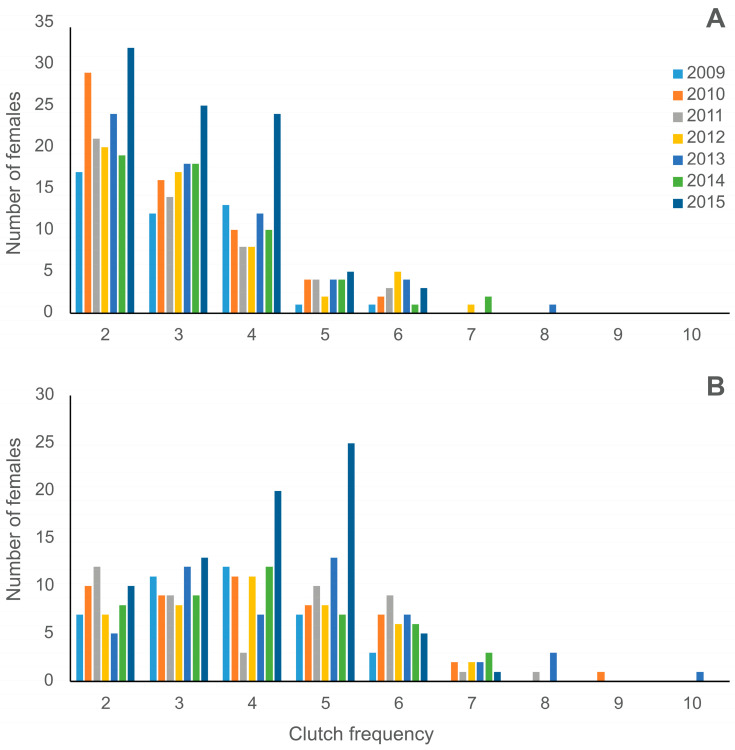
Observed clutch frequency (OCF) (**A**) and estimated clutch frequency from beach monitoring (ECF_BM) (**B**) of loggerhead turtles at the study area in Praia do Forte from the 2009 to the 2015 nesting seasons.

From the 2009 to the 2016 nesting seasons, the OCF for 394 nesting females ranged from 2 to 8 clutches per female (mean 3.1 clutches ± 1.2 SD), and the ECF_BM ranged from 2 to 10 clutches (mean 3.9 clutches ± 1.5 SD; Figure 3).

Based on the combination of data from beach monitoring and satellite telemetry for where the RL was calculated (mean 70.4 days ± 12.8 SD), the ECF_BMST was 5.6 ± 0.7 SD clutches per female (range: 4.2–6.7 clutches per female; Table 1). One turtle observed nesting six times during a season and exhibiting an unusually long RL of 131 days produced an ECF_BMST of 9.8 (Table 1). The RL of this unusual animal is more than 5 SD above the mean of the other animals. Given that we are focused on understanding overall parameters for the nesting aggregation, an outlier among a small number of satellite-tracked turtles could bias the estimates; therefore, this individual was excluded from analyses.

For the selected subset of turtles equipped with transmitters, the average OCF was 4.2 ± 1.3 SD per female (range 2–6 clutches), and the average ECF_BM was 4.5 ± 1.3 SD per female (range 2–6.7 clutches), excluding the two transient turtles (Table 1).

OCF, ECF_BM, and ECF_BMST differed significantly among estimation methods (Kruskal–Wallis test: χ^2^ = 92.54, df = 2, *p* < 0.0001). Pairwise comparisons showed that ECF_BM differed from OCF (*p* < 0.0001), and ECF_BMST differed from both OCF (*p* < 0.0001) and ECF_BM (*p* = 0.000096).

## 4. Discussion

Clutch frequency of loggerhead turtles nesting in Bahia varied depending on the monitoring method used. Using beach monitoring data, the estimated ECF_BM was 3.9 clutches per female. In contrast, when satellite telemetry data were included, the estimated ECF_BMST increased to 5.6 clutches per female, a 44% increase compared to the value derived from beach monitoring alone. This substantial difference highlights the limitations of relying exclusively on traditional beach monitoring data and demonstrates the importance of incorporating complementary techniques to obtain more accurate estimates. This pattern has also been noted in other studies [5,6,8,9,21]. It is important to note that studies have used haul-out (sensor indicating tag is out of the water for an extended period) and/or Fastloc-GPS data to identify additional nesting events [6,7]. Even though our transmitters provided haul-out data, a previous study indicated that the approach we used here is robust to detect the final nesting event before departure from the nesting area [6].

Our standardized effort tagging program, initiated in 2008, enabled a more detailed assessment of clutch frequency (CF) in this population. Based on beach monitoring alone, the observed and estimated number of clutches laid per female varied widely across the population, ranging from 2 to exceptional cases of 9 or 10 clutches. However, integrating these observations with complementary monitoring techniques produced more accurate estimates. The resulting mean CF of 5.6 clutches per female derived from satellite telemetry is therefore considered a more realistic measure of this population’s reproductive output. The estimated increase in CF among loggerhead turtles nesting in Bahia has important implications for population estimates and conservation strategies. This higher CF suggests that female loggerheads lay more nests per season than previously thought, reinforcing the need to revise demographic models and conservation priorities accordingly.

Clutch frequency estimates for loggerhead turtles (*Caretta caretta*) vary significantly across populations globally, reflecting both biological differences and methodological approaches. In Kyparissia Bay, Greece, satellite telemetry revealed a mean clutch frequency of 3.8 nests per female per season [21], notably higher than the 2.2 nests reported from Cyprus using beach patrols [5]. Cabo Verde’s Boa Vista Island—one of the world’s largest rookeries—showed corrected clutch frequencies (i.e., considering females with three or more clutches) that ranged from 3.98 to 4.1 nests per female [12]. In the Southeast USA, values have been revised upward to 4.5–5.4 using telemetry [9] and genetic sampling [22]. Similarly, on Masirah Island, Oman, satellite telemetry indicated a mean clutch frequency of 5.4 nests [23].

The high average number of clutches deposited by female loggerheads in Praia do Forte places this population at the upper end of the reported range for this species. This finding emphasizes the importance of Bahia’s coast as a highly productive nesting area, which may contribute to the population’s resilience against various threats and environmental changes in the Southwest Atlantic [24]. We highlight a possible caveat in satellite-telemetry-derived estimates based on animals first captured only in the first part of the nesting season, as they may not necessarily be representative of the nesting aggregation. Hawksbills nesting in the first portion of the nesting season have higher clutch frequency than the animals arriving later in the season [6]. Clutch frequency may also be influenced by foraging sites, either by food source or distance from the nesting site (energy needed to migrate) [25,26,27].

The major impact of changes in CF estimates is due to its relevance for female abundance estimates. The number of nesting females in a rookery can be estimated, for instance, as follows:

(1) ANF=N/CF

where ANF is the number of annual nesting females, *N* is the annual number of nests laid, and CF is estimated clutch frequency. Given the inverse relationship between CF and ANF, the 44% increase in the CF estimate may represent a ~30% decrease in the ANF. Future population abundance estimates for this population must consider estimates reported here for more accurate assessments.

It is important to note, however, that the telemetry-based sample size was relatively small compared with beach monitoring, reinforcing the need for additional studies—with larger sample sizes and combined methodologies—to refine clutch frequency and other reproductive parameters. In addition to telemetry, genetic analyses can provide an alternative way to refine clutch-frequency estimates. By genotyping embryos or hatchlings from multiple nests, researchers can link all clutches to individual females, including those laid outside the patrol coverage [22]. This approach has improved abundance estimates in other populations [22] and could serve as a valuable complement to telemetry in Bahia and elsewhere. Furthermore, the differences in estimates between approaches highlight the importance of CF reassessment in other subpopulations for broad abundance estimates. Ideally, in a saturation tagging program, researchers should track individuals throughout the nesting season to better assess variation within individuals. Another possible bias could be due to our sampling effort, which was limited to a beach section and specific hours. Even though it was designed to maximize the number of animals captured, it could introduce biases into our estimates. Finally, the implications of this higher clutch frequency extend beyond local population assessments and may affect regional and global conservation strategies for loggerhead turtles. Further research is needed to understand the factors contributing to the higher reproductive output and its long-term effects on population dynamics.

## 5. Conclusions

Our study demonstrates that clutch-frequency estimates for loggerhead turtles in Praia do Forte, Bahia, are substantially higher when satellite telemetry is combined with beach monitoring. The corrected mean of 5.6 nests per female indicates that traditional assessments based solely on beach patrols have underestimated this number. Given that this revised estimate represents an increase of approximately 44% relative to estimates derived from beach monitoring alone, incorporating more accurate demographic parameters can substantially alter estimates of the number of breeding females. Integrating these refined values into regional and global assessments will improve the accuracy of population evaluations and support conservation strategies for loggerheads. Future recommendations include combining alternative methodologies with beach monitoring data to reassess key reproductive parameters in other subpopulations and improve broad abundance estimates.

## Figures and Tables

**Table 1:** Summary of nesting data for the subset of satellite-tracked loggerhead turtles. CCL: curve carapace length; OCF: observed clutch frequency; ECF_BM: estimated clutch frequency based on beach monitoring data; ECF_BMST: estimated clutch frequency based on beach monitoring and satellite telemetry data. Residency length (RL) was calculated as the number of days between the first documented nesting event and the departure date. * Indicates turtles tracked for consecutive nesting seasons, ^t^ indicates transient turtles that were excluded from OCF and ECF_BM calculations. ^#^ Female not included in the mean value calculations (see results).

Turtle ID	CCL (cm)	First Nest Date_BM	Last Nest Date_BM	Departure Date_ST	RL (days)	OCF	ECF_BM	ECF_BMST
1 ^#^	99.2	10/03/2013	02/11/2014	02/11/2014	131	6	9.8	9.8
2 ^t^	92.6	10/19/2013	10/19/2013	12/28/2013	70			5.7
3	102.0	10/21/2013	12/18/2013	01/06/2014	77	4	4.9	6.2
4	102.0	10/22/2013	11/06/2013	12/08/2013	47	2	2.0	4.2
5 ^t^	102.0	10/24/2013	10/24/2013	01/11/2014	79			6.3
6	101.0	11/02/2013	01/03/2014	01/17/2014	76	5	5.2	6.1
7	95.0	10/03/2013	11/06/2013	12/11/2013	69	3	3.3	5.6
8	98.5	10/15/2014	11/17/2014	01/01/2015	78	3	3.2	6.2
9	102.0	10/15/2014	12/26/2014	12/26/2014	72	6	5.8	5.8
10	100.3	10/29/2014	12/27/2014	12/27/2014	59	5	5.0	5.0
11	94.5	11/01/2014	01/25/2015	01/25/2015	85	6	6.7	6.7
12	100.3	11/02/2014	01/07/2015	01/07/2015	66	5	5.4	5.4
6 *	100.0	11/15/2014	12/29/2014	01/11/2015	57	4	4.0	4.8
9 *	104.0	10/26/2017	12/11/2017	12/20/2017	55	3	4.1	4.7
Mean					68.5	4.2	4.5	5.6

## Data Availability

The satellite telemetry data used for the analysis and the beach monitoring data are available upon request.

## Abbreviations

CCL	curved carapace length
CMR	capture-mark-recapture
CF	clutch frequency
ECF	estimated clutch frequency
OCF	observed clutch frequency
ECF_BM	estimated clutch frequency based on beach monitoring
ECF_BMST	estimated clutch frequency based on beach monitoring and satellite telemetry
RL	residency length
RI	remigration interval
